# Challenges in postnatal care provision in Ethiopia

**DOI:** 10.3389/fpubh.2022.922933

**Published:** 2022-08-04

**Authors:** Elias Teferi Bala, Lizeth Roets

**Affiliations:** Department of Health Studies, University of South Africa, Pretoria, South Africa

**Keywords:** postnatal care, challenges in postnatal care, Ethiopia, maternal and child health, quality postnatal care

## Abstract

**Background:**

Most maternal deaths occur during the first 48 h after delivery; thus, a critical time for monitoring possible complications arising from the delivery. Quality postnatal care can contribute to a decrease in maternal mortality and morbidity rates. Despite the importance of postnatal care, it is generally a neglected aspect of maternal and child health services in most developing countries, including Ethiopia.

**Objectives:**

The objective of the study was to describe the challenges experienced by postnatal care providers and coordinators in providing postnatal care in the Ethiopian context.

**Methods:**

A quantitative cross-sectional descriptive study was conducted and data were gathered from 422 postnatal care providers and coordinators during November 2018. A simple random sampling technique was used to select the respondents and the data were gathered through a self-administered questionnaire. The data were cleaned, coded and entered into the Statistical Package for Social Sciences (SPSS) version 21 for analysis. Open-ended questions for qualitative enhancement were open-coded and thematically analyzed.

**Results:**

The findings revealed a lack of physical resources; infrastructure problems; cultural concerns; inadequate capacity building; inaccessibility of health services; unavailability of guidelines; a lack of communication with healthcare users and poor monitoring and evaluation as challenges.

**Conclusion:**

To improve postnatal care in Ethiopia and, ultimately, mother and child health, the challenges experienced by postnatal care providers and coordinators have to be dealt with. A strategic action plan with the active involvement of all stakeholders must be developed and implemented to deal with the challenges and improve postnatal care.

## Introduction and background

Despite the goals set by the United Nations (UN) to reduce maternal mortality by three-quarters from 1990–2015, and the sustainable development goals to reduce maternal mortality in Ethiopia to 199 per 100,000 live births, the rates are still unacceptably high (1, 2). In Ethiopia the maternal mortality rate remained stagnant over the past 18 years with an average of between 412 and 871 per 100,000 live births (3). The postnatal period is an area of concern, since more than 60% of maternal deaths occur during this period and about 45% of them die within 1 day of delivery. The risk of maternal death is the highest close to birth, especially within the first 24 h and then gradually decreases over the subsequent days and weeks. It is estimated that 65% of maternal deaths occur within 1 week of delivery and nearly 80% within 2 weeks of delivery (4–6).

Postnatal care is among the major recommended interventions that can reduce maternal deaths globally, because during this period health professionals can diagnose postpartum problems and potential complications to ensure prompt treatment or interventions (6). Maternal morbidity and mortality can be prevented or reduced significantly if women their families and the community recognize the danger signs and promptly seek healthcare services during the postpartum period (7).

Scientific evidence indicates that the provision and utilization of postnatal care services in many African countries is challenged by factors such as (1) the 40-day period after childbirth that allows mothers and babies to be only indoors for the first month; (2) misconceptions about the importance of postnatal care; (3) the lack of awareness of postnatal care and its benefits; (4) the cost of health services; (5) transport costs; (6) accessibility and the (7) distances to health facilities (8–12). It has also been reported that the attitudes of healthcare providers are a barrier to the utilization of postnatal care in many countries (13, 14).

Despite the existence of evidence on the importance and advantages associated with optimal utilization of postnatal care from different sources, the uptake of these services is still very low and varies across regions and countries (11, 15). In low-income countries, like Ethiopia, the utilization of postnatal care is as low as 19% (3). Hence, the objective of this study was to identify the challenges of postnatal care service provision in Ethiopia to recommend interventions for improvement.

## Methods

### Study setting and period

The study was conducted in Oromia regional state in Ethiopia during the month of November 2018. Administratively, Oromia is divided into 18 zones that are divided into 309 districts (councils), 44 town administrations and 6,881 *kebeles* (subdivisions) (16).

### Study design

A quantitative cross-sectional descriptive study was conducted to identify challenges pertaining to postnatal care service delivery in Ethiopia. Data were gathered from a stratified random sample of a population of 2,925 postnatal care providers (2,865) and 60 coordinators at various health facilities, departments, health centers and hospitals and from district and regional health departments.

### Sample size

A total sample of 422 respondents was determined, using the single population proportion formula *n* = Z (α/2)2^*^P (1-P)/d2

*n* = 1.962 1 (1–0.5)/0.052

*n* = 384

The assumptions under this formula were:

• *n* = sample size

• *Z* (α/2) = the value of normal distribution, representing a confidence level of 95% with a value of 1.96.

• *P* = Proportion of the case

• *d* = Margin of error—considering a non-response rate of 10%, the final sample size was 422. The first assumption was that no studies were conducted on the specific topic and the prevalence is considered to be 50% at 95% confidence interval, with a margin of error (confidence limit) of 5%. The other assumption was that there might be a 10% non-response rate.

### Data collection instrument

A self-developed questionnaire based on a thorough literature review was pre-tested and used for data gathering. Open-ended questions were included for qualitative enhancement to allow for personal opinions and views.

### Data collection

The data collectors received a 3-day training course before the data collection process commenced. The data collectors and researchers distributed an information letter as well as the voluntary consent form among all selected respondents at the health facilities and postnatal care departments. After reading and understanding the information sheet, the volunteers signed the consent form, indicating their willingness to participate. This was followed by questionnaires administration to every consenting participant, requesting them to complete the questionnaire in private in their own time and return the completed questionnaire within 2 days.

### Data quality management

To enhance the validity and reliability of the instrument, a pre-test was conducted among 5% (21 pre-test respondents) of the sample size that had similar characteristics as the study participants but were selected from health facilities and departments located outside the study areas. Data were gathered by ten purposively selected data collectors.

### Data analysis

Data were cleaned, coded and captured from the 422 questionnaires into the Statistical Package for Social Sciences (SPSS) software programme (version 21) for analysis. The findings were summarized and presented in tables and pie-charts, using frequencies and percentages. The responses to open-ended questions were open-coded and thematically analyzed. Although the quantitative data obtained provided valuable insights into the challenges experienced, the narrative data that was thematically analyzed provided rich data to allow for a more comprehensive description of the study findings.

### Ethical considerations

Ethical approval was obtained from the Research Ethics Committee of the Department of Health Studies, University of South Africa (UNISA) to conduct the study. Permission letters to support the study and gain access into the field were received from the respective administrative offices of Oromia Regional State Health Bureau, from each health facility such as health centers, hospitals, district and regional health departments before involving the participants in the study. The participants received an information letter about the research, the objectives of the research and their right not to participate, not to answer a question or to withdraw from the study at any time during data collection without any negative consequences for them.

## Results

### Socio-demographic characteristics

Four hundred and twenty-two respondents agreed to participate and provided completed questionnaires for analysis.

Hundred and forty-two (*f* = 33.6%) respondents were between 30 and 39 years old, 122 (*f* = 28.9%) were between 40 and 49 years and 115 (*f* = 27.3%) were younger than 30 years (refer to Table 1).

**Table 1:** Socio-demographic characteristics of respondents (*N* = 422).

**Variable**	**Category**	**Frequency**	**Percent**
Age	Younger than 30	115	27.3
	30–39	142	33.6
	40–49	122	28.9
	50–59	39	9.2
	60 and older	4	0.9
Gender	Male	174	41.2
	Female	248	58.8
Educational level	Master's	34	8.1
	Bachelor's	263	62.3
	Diploma	125	29.6
Work experience in years	1–10 years	234	55.5
	11–20 years	151	35.8
	21–30 years	37	8.8

More than half of the respondents were females (58.8%) similar to the female/male ratio of the health care providers offering postnatal care in Ethiopia (17).

Two hundred and sixty-three (*f* = 62.3%) were in possession of a bachelor's degree, and 125 (*f* = 29.6%) had a diploma in a healthcare-related field. The evidence indicated that the nurses, midwives and other healthcare providers with advanced qualifications have more advanced knowledge and skills to offer standardized and quality postnatal care (18, 19); therefore, the Ethiopian health system is very privileged (refer Table 1).

Slightly more than half of the respondents (55.5%) had work experience of 10 years or less, with only 35.8% who had between 11 and 20 years of experience in their current position (refer to Table 1). The Ethiopian healthcare workers seemed to have adequate experience to contribute to effective and standard postnatal care provision, as indicated in the literature (20).

**Figure 1:**
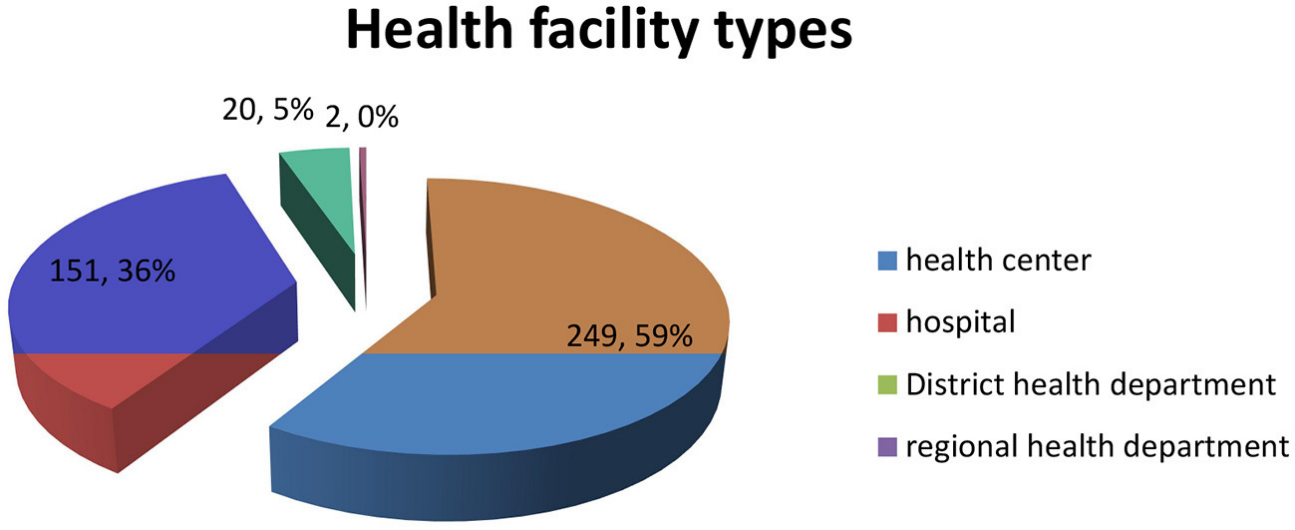
Distribution of respondents per type of health facility.

As indicated in Figure 1, many respondents (59.0%) were working at health centers, 151 (*f* = 36%) were working in district hospitals, and 20 (5%) were working at the district health department. Only two (0.5%) were working at the regional health department.

### Physical resources

Quality, evidence-based and standardized postnatal care can be provided if guidelines are available and used (21). **Guidelines on postnatal** service delivery need to be assessable to all service providers. A matter of concern is that 24.6% (*n* = 104) of respondents do not have immediate access to guidelines related to postnatal care (refer to Table 2). **Inaccessible guidelines** can compromise the postnatal care rendered and lead to differences in the quality of services offered to mothers and their babies (6).

**Table 2:** Postnatal care practices in Ethiopia (*N* = 422).

**Variable**	**Category**	**Frequency (F)**	**Percent (%)**
Availability of separate postnatal unit	Yes	238	56.4
	No	184	43.6
Orientation in postnatal care	Yes	273	64.7%
	No	149	35.3
Competency of the postnatal care providers	Yes	279	66.1
	No	143	33.9
Continuing professional education	Yes	262	62.1
	No	160	37.9
Accessibility to guidelines	Yes	318	75.4
	No	104	24.6
Utilization of guidelines	Yes	311	73.7
	No	111	26.3

Respondents to the open-ended questions confirmed the need for assessable guidelines:

“*Though there are many challenges on postnatal care services, the shortage of guidelines is a serious challenge which compromises the quality of postnatal care provided to mothers and newborns.”*

“*All health facilities rendering postnatal care must use the recommended guidelines to improve the quality of postnatal care.”*

Alarming was that, despite the benefits of using the guidelines, 26.3% (*n* = 111) of the respondents do not use the guidelines related to postnatal care (refer Table 2), possibly with a further negative impact on postnatal service delivery.

The respondents identified (44. 1%) an inadequate budget allocation for postnatal care services, possibly with a negative impact on the quality of postnatal care.

The provision of quality postnatal care can only be achieved when the health facilities are provided with the necessary equipment and supplies (22). However, a participant emphasized the following in the open-ended questions:

“*Health facilities do not provide quality postnatal care as they lack the necessary equipment needed for postnatal care services.”*

“*Postnatal care in Ethiopia is exposed to the shortage of equipment such as diagnostic equipment and* essential drugs.”

### Infrastructure

In order to render quality postnatal care, a specific room or area designated for postnatal care service delivery is needed to ensure that health care providers provide quality postnatal care to postnatal mothers (23). It is therefore of cause for concern that 43.6% of the respondents indicated that there is not a separate room for postnatal care provision (refer Table 2). This finding is very different from a study conducted in Kenya where 78.82% of respondents reported that postnatal mothers were being nursed in units specially designated for them (23).

Mothers who need to travel long distances to use a service need waiting homes where they can stay from the late gestational age to encourage pregnant mothers and their families to use postnatal care services not only in Ethiopia, but also in other African countries (24). The lack of such a facility was elaborated on:

“*The health facilities lack functional maternal waiting homes where mothers rest before and after delivery.”*

Inadequate bed capacity and rooms available after birth possibly leads to the women being discharged 6 h after giving birth (23, 25). Consistently, the participants mentioned the following:

“Some *mothers are discharged early as there are no adequate rooms and beds in the health facilities.”*

Sometimes the lack of bed capacity resulted in mothers and children sleeping on the floor (14, 26), constituting a real risk to their health. This statement is supported by one of the participants:

“*There are occasions where women sleep on the floor as a result of lack of adequate bed (s) in some health facilities*.”

It is essential that there is electricity and clean running water at every health facility if quality health care is to be rendered (26). However, in the Ethiopian context, the participants indicated the following:

“*Many of the health facilities in rural areas use lamps during the night-time to provide skilled delivery and postnatal care services which compromise the quality of services offered to mothers.”*

“*In some health facilities, there is no water supply.”*

### Inaccessible health services

The accessibility of the health service is one of the factors that determine its utilization, since maternal interest alone may not ensure the utilization of the services (27). Similarly, participants in the current study shared the following in this regard:

“*In Ethiopia, though there are improvements in accessibility of the maternal health care including postnatal care, still many women in rural areas do not have access that must be improved.”*

**Inadequate or inappropriate transport and the unavailability of ambulance** services make it difficult for women to reach (28) the health facilities which compromises the utilization of postnatal care. The participants referred to this concern by responding as follows:

“Though *there are improvements in transportation services in Ethiopia still it is a challenge for postnatal care services as many women from rural areas do not have transportation access and are forced to walk on foot for more than 2 h when they seek health services.”*

**Road inaccessibility** is one of the challenges related to transportation services when offering postnatal services (29, 30), which negatively impact on postnatal care in Ethiopia and other countries. The participants mentioned the following in this regard:

“*The unavailability of roads and bad terrain in Ethiopia is a barrier for many women residing in rural areas of Ethiopia that need to be addressed if postnatal care is to be improved.”*

The distance to health facilities is challenging as women residing in remote areas have difficulty in utilizing maternal health services, including postnatal care (31). This is also confirmed by narrative data:

“*Long-distance to the health facility is a barrier for postnatal care utilistion as many women may not seek health care when the health facility is far.”*

### Cultural concerns

**Figure 2:**
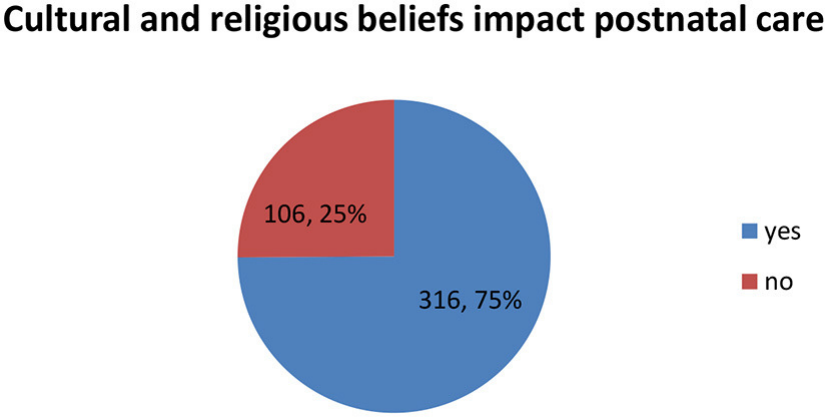
Cultural and religious practices impact postnatal care.

**Cultural practices** have an impact on the health of mothers and babies during the postnatal period (32); therefore, such practices must be identified and attended to when offering the service. In this study 75% (*n* = 316) of respondents were of the opinion that the cultural practices in the community have an impact on both the provision and reception of postnatal care (refer to Figure 2).

Some participants' emphasized the impact of culture:

“*There is no preparation of porridge in the health facilities even the coffee ceremony is not commonly practiced. However, women in Ethiopia like to have those cultural ceremonies.”*

The 40-day period after childbirth that allows mothers and babies to be indoors for the first month, has a negative impact on the utilization of postnatal care. In some cultures the postnatal mother wants to attend to cultural practices in their homes (8), as indicated by some participants:

“*Some postnatal women want to remain at home during the postnatal period to practice their beloved culture.”*

**Figure 3:**
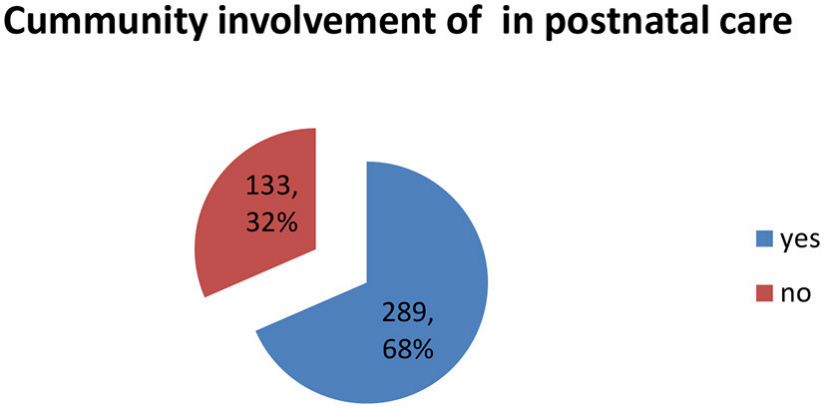
Community involvement.

Despite the fact that health service programmes run successfully when community members are involved, as described by Babalola, Van Lith, Mallalieu, Packman, Myers, Ahanda, et al. (33), 32.0% (*n* = 133) of respondents did not involve community leaders in planning and providing postnatal care services (refer to Figure 3).

### Lack of communication

Professional orientation before offering a service is essential to ensure quality care (23); thus, orientation given by experienced postnatal care providers or unit coordinators will make a significant contribution to quality postnatal care. However, in the Ethiopian context only 64.7% (*n* = 273) of respondents indicated that they have received orientation on postnatal care.

Effective telephone communication between the postnatal mother or her family to notify midwives, health extension workers or other health professionals when in need of postnatal care can increase the utilization of postnatal services (34). Participants in this study reported the following in this regard:

“*Many women and their families in Ethiopia do not know whom to call and where to go when they need postnatal care or develop any health problem.”*

### Inadequate capacity building

Incompetent postnatal care providers pose a challenge for postnatal service delivery, as competency is a pre-requisite for standardized quality postnatal care (21, 35). The study findings, however, revealed that 33.9% (*n* = 143) of postnatal care providers indicated that they **lack the competency** to provide quality postnatal care (refer to Table 2).

The Ethiopian Federal Ministry of Health (FMOH) recommends continuing professional development to ensure that healthcare providers possess the competencies required to practice safely, effectively and to provide quality healthcare services. However, this study revealed that only 62.1% (*n* = 262) participated in continuous professional education to ensure competency while working in the postnatal care unit (refer Table 2).

Postnatal care providers need to participate in continuing professional development to update their knowledge and skills for providing quality and standardized postnatal care (36). Participants confirmed this requirement and reported as follows:

“*Postnatal care providers should attend continuing professional development frequently to update their skills if postnatal care services are to be improved.”*

“*Postnatal care providers who have no continuing professional development have difficulty in rendering quality services.”*

### Poor monitoring and evaluation

Effective postnatal care monitoring and evaluation mechanisms are of great importance to the assessment of the quality of postnatal care offered to mothers and their babies; responses to the interventions provided to mothers and babies, and whether the postnatal care services led to the objectives being achieved (37). Postnatal services need to be monitored and evaluated at all levels where postnatal service provision and coordination is carried out, as indicated by participants in the current study:

“*In most of the health facilities, monitoring, and evaluation of postnatal care services are poor. However, as it is an integral component of the postnatal services it needs to be improved in Ethiopia.”*

“*There should be strong monitoring and evaluation if postnatal care is to be improved.”*

## Discussion

The challenges identified by respondents in the Ethiopian context that can influence the provision and utilization of postnatal care were similar to the challenges identified in other studies (11, 38). Postnatal service delivery will not improve in the absence of research into human resources, physical resources, financial resources and equipment and supplies (22, 23).

Challenges pertaining to the lack of physical resources due to an inadequate budget namely, electricity constrains, limited water supply, inadequate equipment and supplies and limited bed capacity and rooms available after birth are posing a challenge to postnatal care in various regions of the world (14, 22, 24, 26).

Challenges pertaining to the infrastructure of healthcare facilities and accessibility of these facilities in terms of poor road infrastructure and transport challenges are documented challenges in developing countries (28–31), despite being a requirement for postnatal care improvement (23, 36).

Findings from this study indicated the inaccessibility of health services as a challenge for quality postnatal care. Similar evidence is documented in literature studies (39, 40).

Quality, evidence-based and standardized postnatal care can only be provided if guidelines are made available and used by all healthcare workers. To prevent unorganized and fragmented postnatal care being provided to postnatal mothers and their newborns (34).

Effective telephone communication between the healthcare providers and the community (23, 34) and a quality monitoring and evaluation system are essential components of quality postnatal care delivery.

From the literature, it is evident that numerous attempts were made to improve postnatal care and postnatal care service delivery. Despite all the efforts made, it remains a challenge as utilization remains low. An action plan involving all stakeholders in the development thereof (mothers, healthcare professionals, policy-makers) is needed to facilitate the implementation of strategies and guidelines. This will encourage ownership of the action plan, including providing guidance on who needs to take responsibility for which actions within a very specific timeline. In this way progress can be monitored and accessed and a plan can be adopted or adapted to suit changing healthcare environments and context.

## Conclusion

The study revealed there is a lack of physical resources, infrastructure problems, cultural concerns, inadequate capacity building, inaccessibility of health services, unavailability of guidelines, a lack of communication with healthcare users and poor monitoring and evaluation as challenges which negatively impact on the provision and utilization of postnatal care services similar to other African countries. Healthcare professionals in the African context need to take responsibility and provide a realistic solution in the form of action plans to facilitate the process, ultimately improving maternal and newborn health. As part of the action plans, maternal dignity, respect and satisfaction can get due attention to ensure that the service is utilized in future.

## Data availability statement

The raw data supporting the conclusions of this article will be made available by the authors, without undue reservation.

## Ethics statement

The studies involving human participants were reviewed and approved by University of South Africa (UNISA). The patients/participants provided their written informed consent to participate in this study.

## Author contributions

All authors listed have made a substantial, direct, and intellectual contribution to the work and approved it for publication.

## Conflict of interest

The authors declare that the research was conducted in the absence of any commercial or financial relationships that could be construed as a potential conflict of interest.

## Publisher's note

All claims expressed in this article are solely those of the authors and do not necessarily represent those of their affiliated organizations, or those of the publisher, the editors and the reviewers. Any product that may be evaluated in this article, or claim that may be made by its manufacturer, is not guaranteed or endorsed by the publisher.
